# Genomic dissection of the seed

**DOI:** 10.3389/fpls.2014.00464

**Published:** 2014-09-12

**Authors:** Michael G. Becker, Ssu-Wei Hsu, John J. Harada, Mark F. Belmonte

**Affiliations:** ^1^Department of Biological Sciences, University of Manitoba, Winnipeg, MBCanada; ^2^Department of Plant Biology, University of California Davis, Davis, CAUSA

**Keywords:** *Arabidopsis*, next generation sequencing, oilseed, RNA seq, seed, soybean, transcriptome

## Abstract

Seeds play an integral role in the global food supply and account for more than 70% of the calories that we consume on a daily basis. To meet the demands of an increasing population, scientists are turning to seed genomics research to find new and innovative ways to increase food production. Seed genomics is evolving rapidly, and the information produced from seed genomics research has exploded over the past two decades. Advances in modern sequencing strategies that profile every molecule in every cell, tissue, and organ and the emergence of new model systems have provided the tools necessary to unravel many of the biological processes underlying seed development. Despite these advances, the analyses and mining of existing seed genomics data remain a monumental task for plant biologists. This review summarizes seed region and subregion genomic data that are currently available for existing and emerging oilseed models. We provide insight into the development of tools on how to analyze large-scale datasets.

## INTRODUCTION

With the world population expected to reach over 9 billion by the middle of the 21st century, one of the biggest challenges facing humanity will be the production of sustainable food supplies (Godfray et al., 2010; Cleland, 2013). To accommodate world food demands, it is estimated that crop production will need to double without increasing current agricultural land use (Foley et al., 2011; Tilman et al., 2011; Ray et al., 2013). Since the direct consumption of seeds and their use as animal feed account for more than 70% of the human diet (Sreenivasulu and Wobus, 2013), recent discussions on food security have turned to enhancing crop production through seed genomics. Seed genomics is the study of genomes and the expression of genes that are required to make a seed. This includes the spatial and temporal expression and regulation of all genes active during seed development. While classical breeding strategies have proven to be effective in producing more robust and productive plant cultivars, they can be complemented and greatly improved through the utilization of genomics-based knowledge (Tester and Langridge, 2010; Feuillet et al., 2011; Langridge and Fleury, 2011).

A seed is formed upon fertilization of the female gametophyte and early stages of development involve the deterioration of maternal gametophytic structures and the establishment of the sporophyte. Seed development is initiated by a double fertilization event that results in a seed that can be divided into three distinct regions: the embryo, the endosperm, and the seed coat (SC; Ohad et al., 1999; Le et al., 2007). In the first fertilization event, a sperm, and egg cell nucleus fuse, resulting in a zygotic embryo. The embryo is part of the next sporophytic plant generation. The endosperm results from the second fertilization event between the sperm and central cell, and it will serve to support the embryo during the early stages of seed development and/or seedling growth. Finally, the seed coat (SC) is of maternal origin and is derived from the integuments that form during ovule development. The SC transfers assimilates from the maternal plant and serves to protect the embryo throughout seed development. Further, the developmental programs that underlie seed development can be divided into two distinct phases. First, during morphogenesis, the body plan of the embryo is established and the nuclei of the endosperm proliferate. Second, during the maturation phase, large shifts in gene activity are observed across all three regions of the seed, initiating the accumulation of storage materials that help to protect the embryo in preparation for desiccation.

**FIGURE 1:**
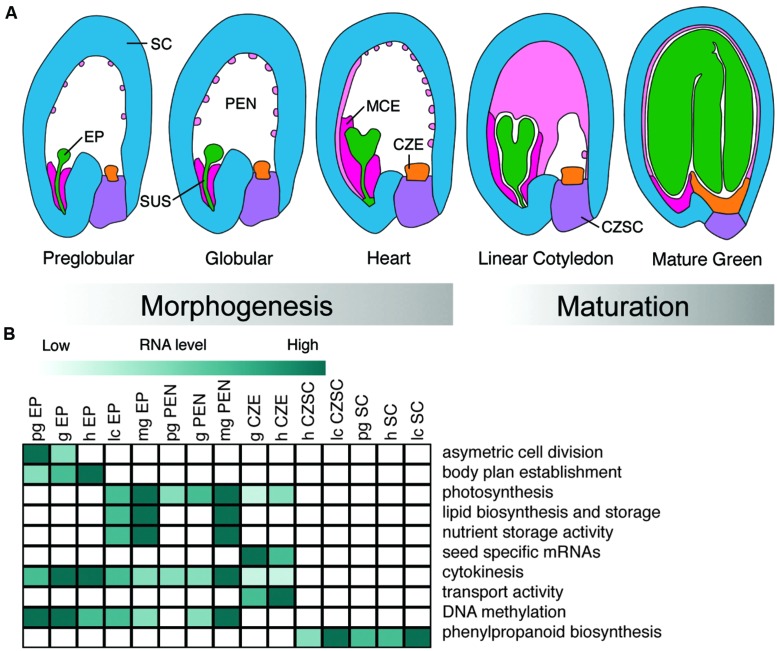
**Development and biological functions of *Arabidopsis* seed subregions. (A)** Representation of seed subregions in *Arabidopsis* from the preglobular to mature green stages of development. Green, embryo proper (EP); dark pink, micropylar endosperm (MCE); light pink, peripheral endosperm (PEN); orange, chalazal endosperm (CZE); purple, chalazal seed coat(CZSC); blue, seed coat (SC). **(B)** Heat map visualization of representative Gene Ontology terms, biological processes, and metabolic pathways found in different subregions of the seed discussed in the review. Preglobular (pg); globular (g); heart (h); linear cotyledon (lc); mature green (mg). Dark green color represents activity in a particular subregion of the seed over developmental time.

We can further dissect seed regions into subregions. In numerous plants including *Arabidopsis*, the zygote differentiates into the embryo proper, which will become cotyledonous and eventually form the vegetative plant, and the suspensor, which acts to facilitate communication between the embryo proper and surrounding seed regions. The endosperm develops into three distinct subregions: the micropylar endosperm (MCE, proximal to the embryo), the peripheral endosperm (PEN), and the chalazal endosperm (CZE, distal to the embryo; Brown et al., 2003). The maternally derived SC can be divided into two subregions, the chalazal seed coat (CZSC), and distal SC (**Figure 1A**). Depending on the model seed, these subregions can further be divided into tissue and cell types.

This review focuses on the genomic analysis of seed regions and subregions using established and emerging plant models. We discuss how genomics has been used successfully to study the development of the embryo, endosperm, and SC regions of the seed, and how new cutting-edge tools can be used to further dissect every cell and tissue of the seed into subregions for further interrogation. Finally, we present tools on how to analyze large-scale transcriptome datasets.

## CHARACTERIZING THE SEED TRANSCRIPTOME

In the current genomics era we have uncovered a number of developmental and regulatory pathways responsible for making a seed. However, we still have yet to fully understand all of the mechanisms responsible for the coordination of gene activity underlying the sophisticated development of all seed regions and subregions. Many regulatory mechanisms surrounding primary and secondary metabolism, hormone regulation, gene imprinting, transcriptional-, translational-, and post-translational regulation all operate in concert to mediate the complex processes occurring during seed development. These processes are under the regulation of 100s and 1000s of genes that are often obscured by genetic redundancy and thus difficult to identify using traditional forward genetics screens (Curtin et al., 2011). Arguably, the best way to investigate coordinated events such as cell fate specification, differentiation, and morphogenesis of the developing seed is by monitoring the expression of large gene sets with high throughput genomics-focused microarray and sequencing strategies. Bioinformatic analyses can then be used to identify transcriptional networks and key regulators of seed development.

As Next Generation Sequencing experiments such as deep genomic sequencing, RNA-, small RNA-, and DNA methylome-sequencing become commonplace in the laboratory and as sequencing technologies continue to evolve, the challenge faced by the scientific community is no longer the acquisition of data, but rather compiling and analyzing the data. Publicly available databases like NCBI (The National Center for Biotechnological Information ^1^), GEO (Gene Expression Omnibus ^2^), and SRA (Sequence Reads Archive ^3^) contain large amounts of DNA microarray and nucleic acid sequence data that can be queried and mined to provide answers to challenging biological questions about the seed.

### USING DNA MICROARRAYS TO PROFILE THE SEED

The Affymetrix ATH1 GeneChip microarray was one of the most widely used tools to profile the *Arabidopsis* transcriptome, and it was used to investigate numerous processes underlying the seed including gibberellin response (Ogawa et al., 2003), response to abscisic acid (Nishimura et al., 2007), seed dormancy (Finch-Savage et al., 2007), seed imbibition (Nakabayashi et al., 2005; Preston et al., 2009), seed germination (Dean Rider et al., 2003; Penfield et al., 2006; Dekkers et al., 2013), and development (Day et al., 2008; Le et al., 2010; Dean et al., 2011; Belmonte et al., 2013; Khan et al., 2014).

Le et al. (2010) published the *Arabidopsis* seed transcriptome at seven stages of development from ovule to seedling and identified putative regulators of seed development. At each stage of development, between 8779 and 13,722 distinct mRNAs were detected at the level of the GeneChip with 15,563 unique transcripts detected over all stages of seed development. Of these, only 2% (289) of the transcripts were considered seed-specific with the vast majority being specific to a given stage of development (e.g., globular-cotyldeon). Of these seed-specific genes, 17% coded for transcription factors (TFs) and contained known regulators of seed development, such as *LEAFY COTYLEDON1* (*LEC1*), *LEAFY COTYLEDON2* (*LEC2*), *FUSCA3* (*FUS3*), and *MEDEA* (Le et al., 2010).

Similar analyses were conducted for developing soybean seed from five developmental time points ranging from mid-maturation through seed desiccation (Jones et al., 2010). This study noted an increase in TF activity late in seed development. TFs accumulating late in development included those involved in ethylene and auxin responses, as well as genes that were largely uncharacterized in soybean. Orthologous genes in *Arabidopsis* and rice suggest these genes are involved in processes such as abscisic acid and gibberellic acid signaling, sugar and nitrogen metabolism, and germination.

### PROFILING THE SEED USING LASER MICRODISSECTION COUPLED WITH MICROARRAYS

Traditional studies that isolated seed regions like the embryo, endosperm, and SC for seed genomics used forceps or fine needles. The lack of precision of these manual techniques makes it nearly impossible to isolate individual regions without contamination from neighboring cells or tissues. These challenges limit the resolution of genomics research and dilute low abundant transcripts that may otherwise be detected using more sophisticated dissection methods. Regardless of the dissection tool used, the advancement of genomics-based seed research relies on contamination-free isolation of the cells and tissues of interest.

Currently, the most successful way to dissect regions and subregions of the seed for genomics studies without contamination of other cells types is through laser microdissection (LMD) technologies (Khan et al., 2014). Whole-seed mRNA profiling experiments provided some of the most informative seed genomic data across developmental time for *Arabidopsis* and soybean, but the application of LMD to these seeds provided higher resolution and more sensitive profiles of gene activity in developing seed. For example, Casson et al. (2005) dissected the *Arabidopsis* embryo to study mechanisms associated with apical / basal polarity. This study detected expression of ∼65% of the 22,810 probe sets on the ATH1 array during the early stages of embryo development. Characterization of the spatial and temporal expression of 220 genes known to cause defects in embryo development when mutated, including *PASTICCINO1*, *PINOID*, *PIN-FORMED3*, and *PIN-FORMED4* during embryo development provided insight into their control. Further, several of these genes are being used as markers for the embryo.

The endosperm has been a difficult seed region to study using transcriptome analysis given that the endosperm subregions are not easily isolated. LMD has proven to be an effective and contamination-free technique to isolate the individual subregions of the endosperm for transcriptional profiling (Day et al., 2008). An initial study identified 800 genes, 27 encoding TFs that are preferentially expressed during early endosperm development. Biological processes associated with the progression and control of the cell cycle, DNA processing, chromatin assembly, protein synthesis, cytoskeleton- and microtubule-related processes, and cell/organelle biogenesis were all predicted to characterize endosperm proliferation and cellularization.

The most comprehensive developmental series of any seed was recently published by Belmonte et al. (2013) with the goal of identifying all of the genes and defining the gene regulatory networks responsible for guiding seed development. Thirty-six seed subregions across five developmental stages revealed complex dominant patterns of gene activity in both space and time in *Arabidopsis* (Belmonte et al., 2013; data available at seedgenenetwork.net). The combination of LMD and the ATH1 GeneChip identified at least 17,594 distinct mRNAs that are detectable during seed development and 1,316 of those mRNAs are specifically expressed in the *Arabidopsis* seed compared to vegetative and reproductive tissues. Similar data describing mRNA profiles at high spatial resolution are also available for soybean from experiments that used the Affymetrix soybean GeneChip to analyze 40 subregions across four developmental stages (Le et al., 2007; data available at seedgenenetwork.net). The reader is referred to Nelson et al. (2006) and Day et al. (2007) for reviews on methods and protocols used for LMD of plant tissues (**Figure 1B**).

### USING NEXT GENERATION SEQUENCING TO PROFILE THE SEED

There are a number of advantages to NGS sequencing technology when compared to DNA microarrays: (i) the ability to detect low abundance transcripts, (ii) the identification of novel alternatively spliced isoforms of mRNAs, (iii) little requirement for *a priori* knowledge of the organism, (iv) increased sensitivity in the detection of differentially expressed genes, (v) more reproducible results, and (vi) the ability to compare expression profiles between distantly related organisms. For example, RNA sequencing facilitated the study of oil accumulation in four non-model oilseeds (or “emerging models”): castor (*Ricinus communis*), rapeseed (*Brassica napus*), burning bush (*Euonymus alatus*), and nasturtium (*Tropaeolum majus*; Troncoso-Ponce et al., 2011). These species differ in their location for oil deposition, triacylglycerol composition and content. Analysis of the data revealed a core set of well-conserved enzymes involved in triacylglycerol production that exhibit similar temporal expression patterns in all species, suggesting a conserved evolutionary relationship in the production of seed oil. Putative regulators and mediators of oil production in *Arabidopsis* were identified and an online resource, “ARALIP ^4^,” was established to facilitate utilization of these data. It is important to note that while NGS has several advantages over microarray technology, the detection of low abundant transcripts as well the detection of alternative splice sites is largely dependent on the depth of sequencing and should be carefully considered during the design of the experiment.

**FIGURE 2:**
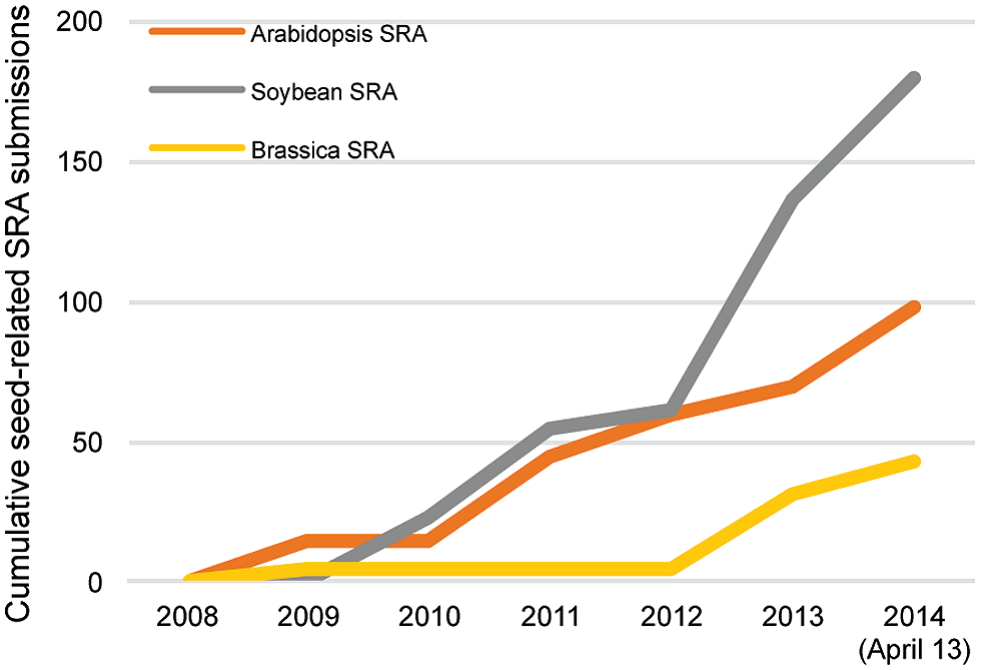
**Cumulative seed related Sequence Read Archive (SRA) submissions in *Arabidopsis* (orange), Brassica (yellow), and soybean (gray) from 2008 to 2014 (April 13) through the National Center for Biotechnology Information**.

Many other RNA sequencing studies of seed genomics have focused on soybean, largely because of its global economic importance. An indication of this emphasis is that seed-related submissions of soybean RNA sequencing data to the SRA and NCBI databases nearly double those of *Arabidopsis* (**Figure 2**). This has produced several large datasets for soybean seed development. Two particular studies stand out, one that profiled the whole soybean seed at seven time points between 10 and 42 days after fertilization (Severin et al., 2010), and an independent study focusing on whole soybean seeds at 15–65 days after fertilization (Chen et al., 2012). These studies showed that 49,151 transcripts are detected during seed development, ∼12,000 mRNAs more than the 37,500 transcripts represented on the current soybean Affymetrix array. Furthermore, 9930–14,058 (Severin et al., 2010) and 11,592–16,255 (Chen et al., 2012) transcripts are differentially expressed compared to the earliest stage of seed development. Both of these studies provide examples of how RNA sequencing data can be mined using a range of bioinformatics approaches including gene ontology term enrichment and co-expression analyses.

Next Generation Sequencing also provides an effective method for the characterization of small RNA (sRNA) populations within the developing seed. Two classes of sRNAs highly expressed within seed tissues are microRNAs (miRNAs) and small interfering RNAs (siRNAs). miRNAs are ∼21 nucleotide, single-stranded, non-coding RNAs that mediate the degradation or translational inhibition of target mRNAs with complementary nucleotide sequences (Chen, 2012). siRNAs, derived from double-stranded RNA, cause the degradation of target mRNAs and carry out *de novo* deposition of repressive chromatin marks and will be discussed later in this review.

Much of the recent work profiling sRNAs during seed development focus on economically important emerging models. Two independent studies examined sRNA populations in soybean, focusing on the identification of miRNAs active during development and their putative targets (Song et al., 2011; Shamimuzzaman and Vodkin, 2012). Of the miRNA targets identified, 50% (Song et al., 2011) and 82% (Shamimuzzaman and Vodkin, 2012) were TFs, including auxin response factors and growth regulating factors. Eleven annotations were found in both datasets including Argonaute Protein, Auxin Response Factor, Growth Regulating Factor, HD-ZIP TF, No Apical Meristem protein, TCP Family TF, and Nuclear Factor YA. These studies also report an increase in mRNA target diversity late in development, suggesting miRNAs have a role in the shift into maturation, which agrees with data from earlier work done with *Arabidopsis* (Tang et al., 2012).

Huang et al. (2013) characterized *B. napus* sRNA populations in whole seeds at nine time points in development and in dissected endosperm, embryo, and SC at three of those stages. Similar to *Arabidopsis* and soybean, the authors suggest that miRNAs have a role in controlling seed maturation. In addition, 279 miRNAs were identified that had been previously reported, including 182 in *Arabidopsis* and 56 in soybean. Also in *B. napus*, Zhao et al. (2012) characterized miRNA populations in high- and low-oil content seeds, and they identified putative miRNA regulators of oil metabolism.

Several databases for miRNAs and their mRNA targets are available to the researcher. Currently 427, 573, and 92 mature miRNA sequences for *Arabidopsis*, soybean, and canola, respectively, have been deposited in miRBase, an online database for published miRNA sequences ( ^5^Kozomara and Griffiths-Jones, 2014). Another database, MiRTarBase ( ^6^Hsu et al., 2014) contains experimentally confirmed miRNA-target interactions (Hsu et al., 2014). In addition, MiRFANs ( ^7^Liu et al., 2012) stores miRNA functional annotations specifically for *Arabidopsis*, and it includes an analysis toolbox.

The production of data from Next Generation Sequencing studies is providing the scientific community with vast amounts of genomic data that can be mined to answer many important biological questions about the seed. Dramatic improvements to seed transcriptome experiments, including enhanced sequencing chemistries and better bioinformatics tools should provide the necessary tools and data required to answer these questions. With Next Generation Sequencing, subtle changes to the transcriptome can now be detected with high confidence and exploited to identify most of the genes and gene products responsible for seed development.

## GENOMICS OF EMBRYO DEVELOPMENT

Embryogenesis is the developmental period during which the zygote differentiates into the mature embryo. Embryo development can be divided temporally into two phases, morphogenesis and maturation (Goldberg et al., 1994). During the morphogenesis phase, the diploid zygote derived from fertilization of the egg cell by a sperm cell undergoes an asymmetric cell division, producing the apical and basal cells (Lau et al., 2012). In many plants, the apical cell gives rise to most of the embryo proper. The basal cell develops largely into the suspensor, although the uppermost suspensor cell divides to form the hypophysis that will become the quiescent center of the root apical meristem and the central root cap cells of the embryo proper. Development of the embryo proceeds along two primary axes. Along the apical-basal axis, the embryo becomes sequentially partitioned into specific pattern elements that become the cotyledons, shoot apical meristem, hypocotyl, root, and root apical meristem. The embryo proper also becomes compartmentalized along its radial axis to generate the embryonic tissue systems: procambium, ground tissue, and protoderm. The suspensor is an ephemeral structure of the embryo that serves a structural role by pushing the embryo proper into the nutrient-rich endosperm and a physiological role by transferring nutrients and growth factors to the embryo proper at early developmental stages (Kawashima and Goldberg, 2010).

As the embryo transitions from the morphogenesis to the maturation phase, morphogenetic processes, including cell division, become largely repressed (Harada, 1997; Vicente-Carbajosa and Carbonero, 2005). During the maturation phase, the embryo acquires the ability to withstand stresses imposed by desiccation that occur late in seed development and accumulates storage proteins, lipids, and/or carbohydrates to massive amounts, causing the embryo to grow as a result of cell expansion. The storage macromolecules serve as a nutrient source for the developing seedling during post-germinative development. By the end of the maturation phase, the embryo is quiescent metabolically and arrested developmentally, and it remains in this state until conditions appropriate for germination and post-germinative development are perceived.

### CONTRIBUTIONS OF THE MATERNAL AND PATERNAL GENOMES TO EARLY EMBRYO DEVELOPMENT

The zygote represents the first stage of the morphogenesis phase, and two studies have addressed the question of when the zygotic genome becomes active transcriptionally following fertilization of the egg cell. In animals, early embryonic development is regulated by maternal mRNAs deposited in the egg prior to fertilization, and the zygotic genome becomes transcriptionally active several cell cycles after fertilization (Tadros and Lipshitz, 2009). The maternal-to-zygotic transition was analyzed in *Arabidopsis* by sequencing RNAs from early stage embryos derived from crosses between plants of different ecotypes and using single nucleotide polymorphisms to distinguish mRNAs derived from maternal and paternal alleles. Autran et al. (2011) reported that the majority of mRNAs in an *Arabidopsis* embryo at the two to four cell embryo proper stage are from the maternal genome, although approximately 10% of mRNAs are encoded by paternal alleles at this early stage. The paternal contribution to the mRNA population increased to 36% by the globular stage, which was interpreted to represent a gradual activation of the paternal genome. Paternal genome activity is maternally regulated through epigenetic mechanisms involving RNA-dependent DNA methylation, KRYPTONITE-mediated histone methylation, and CAF-1 complex-induced histone exchange (Autran et al., 2011). By contrast, a separate study of the maternal-to-zygotic transition reported that maternal and paternal genomes contribute almost equally to the transcriptomes of *Arabidopsis* embryos at the earliest stages of embryogenesis (Nodine and Bartel, 2012). Many mRNAs that are undetectable in the egg and sperm constitute the top 50% most abundant mRNAs in one or two-cell embryos, suggesting that the zygotic genome is activated immediately after fertilization and plays a major regulatory role during early embryogenesis. Discrepancies between the findings of these two studies may have resulted from the use of different *Arabidopsis* ecotypes by the two laboratories (Baroux et al., 2013). Alternatively, the high proportion of maternally derived mRNAs may have resulted from contamination of embryo samples by mRNAs from the SC that is entirely of maternal origin (Nodine and Bartel, 2012). Nevertheless, both studies demonstrated that the maternal-to-zygotic transition occurs at the earliest stage of embryo development in *Arabidopsis*.

### ROLE OF microRNAs IN THE TRANSITION FROM THE MORPHOGENESIS TO MATURATION PHASE

The transition from the morphogenesis to the maturation phase represents a major shift in the developmental programs that occur during embryogenesis (Harada, 1997; Vicente-Carbajosa and Carbonero, 2005). The transcriptomes of *Arabidopsis* embryos that were isolated from the seed by LMD or hand dissection were profiled at several stages of development (Xiang et al., 2011; Belmonte et al., 2013), and these studies demonstrated that gene expression changes dramatically as embryos transition into the maturation phase. For example, the vast majority of mRNAs that accumulate in the embryo proper at a specific stage of development do so at the maturation phase. This gene set is enriched for those involved in maturation processes, including mRNAs encoding storage proteins, oilbody proteins, and proteins involved in lipid storage.

microRNAs play a critical role in controlling the transition from the morphogenesis to the maturation phase (Nodine and Bartel, 2010; Willmann et al., 2011). The role of miRNAs in controlling the transition from morphogenesis to maturation phase was revealed by studies of mutations affecting *DICER-LIKE1* (*DCL1*), which encodes an enzyme required for miRNA biosynthesis. Early in embryo development, loss-of-function *dcl1* mutants display abnormal cell division patterns in the hypophysis, a cell that will become incorporated into the root apical meristem, and in subprotodermal regions of the embryo. These finding were interpreted to suggest that miRNAs are required for embryo patterning events that occur during the morphogenesis phase (Nodine and Bartel, 2010; Willmann et al., 2011). Transcriptome analyses showed that mRNAs that normally accumulate specifically during the maturation phase, including those encoding storage proteins, oil body proteins, lipid biosynthesis enzymes, and several transcriptional regulators of the maturation phase, accumulate prematurely in *dcl1* mutant embryos. By contrast, two TFs, ASIL1, and HDA6/SIL1, that normally repress maturation genes after germination were downregulated in *dcl1* mutants (Willmann et al., 2011). These results, along with the finding that chloroplast maturation occurs earlier in *dcl1* mutant than wild-type embryos, were interpreted to indicate that miRNAs are required to repress maturation processes during the morphogenesis phase and that the precocious onset of the maturation phase in *dcl1* mutants causes defects in pattern formation. In particular, one set of miRNAs and their target mRNAs were implicated to mediate temporal control of the maturation phase. In *dcl1* mutants, disruption of *miR156* accumulation causes the premature upregulation of two differentiation promoting TFs, SPL10, and SPL11, and experiments analyzing the effects of altering *SPL10* and *SPL11* expression suggested that they are at least partially responsible for repressing the maturation processes early in embryogenesis. A different miRNA, *miR166*, has been shown to repress genes expressed specifically during the maturation phase in vegetatively growing plants (Tang et al., 2012). Together, these observations suggest miRNAs play critical roles in controlling embryonic processes.

### MATURATION GENE REGULATORY NETWORKS

**FIGURE 3:**
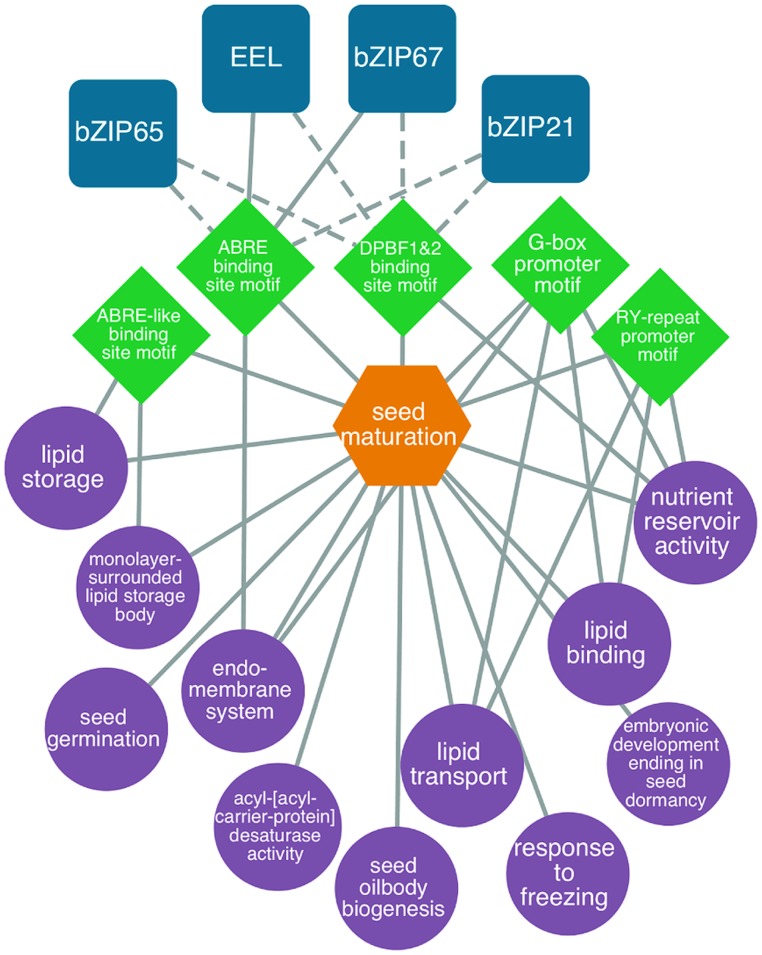
**Predicted bZIP-regulated seed maturation network**. bZIP TFs (blue squircles) are predicted (dashed lines) or known (solid lines) to bind to DNA sequence motifs (green diamonds) within the 1 kb upstream region of the transcription start site in genes associated with enriched GO terms like lipid storage, nutrient reservoir activity and seed oilbody biogenesis (*P* < 0.001, hypergeometric distribution, purple circles). Genes associated with the network are co-expressed during seed maturation (orange hexagons). Modified from Belmonte et al. (2013).

Several studies have focused on understanding the gene regulatory networks that operate during the maturation phase of seed development (reviewed by Santos Mendoza et al., 2005; Gutierrez et al., 2007; Braybrook and Harada, 2008; Holdsworth et al., 2008; Suzuki and McCarty, 2008; Junker et al., 2010). To gain insight into embryo maturation gene regulatory networks, Belmonte et al. (2013) identified DNA sequence motifs that are overrepresented in the 5′ flanking regions of a set of genes that are expressed in embryos specifically during the maturation phase. TFs that are known or predicted to bind these overrepresented DNA sequence motifs were also identified, permitting a putative gene regulatory network to be created. The network included a number of *cis*-acting DNA elements that have been shown previously to regulate genes expressed during the maturation phase, including the *ABRE*, *ABRE-like*, *DPBF1*, *DPBF2*, and *RY* motifs. Identified among the TFs known to bind these motifs were EEL and bZIP67, which are known to regulate genes during the maturation phase. An example of a maturation gene regulatory network is presented in **Figure 3** and a description of the construction of gene regulatory networks is presented in “Identifying regulatory networks required to program the *Arabidopsis* seed” below.

Studies to characterize regulators of the maturation phase have focused on the *Arabidopsis* LEC1, LEC2, FUS3, and ABI3 TFs (Koornneef et al., 1984; Meinke, 1992; Keith et al., 1994; Meinke et al., 1994; West et al., 1994). LEC1 is a HAP3 (a.k.a. NF-YB) subunit of the CCAAT-binding (NF-Y) TF (Lotan et al., 1998), whereas LEC2, FUS3, and ABI3 are B3-domain TFs (Giraudat et al., 1992; Luerssen et al., 1998; Stone et al., 2001). The central roles of these maturation TFs in controlling embryo and seed development was established initially through investigations of mutations in these genes. Loss-of-function mutations in these maturation TF genes cause embryo lethality or the ablation of embryo parts, because mutant embryos are intolerant of desiccation and storage protein and lipid accumulation is defective. Ectopic expression of these maturation TF genes induces somatic embryo development, fatty acid biosynthesis, oil body accumulation and storage protein biosynthesis in vegetative cells (Parcy et al., 1994; Lotan et al., 1998; Kagaya et al., 2005a; Santos Mendoza et al., 2005; Mu et al., 2008; Stone et al., 2008; Feeney et al., 2013).

The maturation TFs LEC1, LEC2, FUS3, and ABI3 are involved in complex and redundant regulatory interactions during embryo development (reviewed by Braybrook and Harada, 2008; Junker et al., 2010). Genetic and molecular experiments have shown that LEC1 functions upstream of LEC2, FUS3, and ABI3 and, therefore, is likely to act at or near the top of the regulatory hierarchy controlling maturation (Kagaya et al., 2005b; To et al., 2006). Redundancy is observed in interactions among the other maturation TFs that is dependent on their spatial location in the embryo (To et al., 2006). For example, the *FUS3* gene is regulated by LEC1, LEC2, and ABI3 in cotyledons, by LEC2 and ABI3 in the embryonic axis, and by LEC2 and FUS3 in the root tip. Together, the results suggest that these maturation TFs play key but complex roles in the regulatory network controlling the maturation phase of seed development.

In recent years, initial dissection of the maturation gene regulatory network has occurred through the genome-wide identification of target genes that are directly regulated by the maturation TFs. Direct target genes are generally defined as those that are bound by a TF, as determined by chromatin immunoprecipitation experiments, and that are regulated by that TF. Genes that are up- and downregulated by a TF are often identified by comparing their mRNA levels in embryos with a mutation in the TF gene versus wild type. Alternatively, regulated genes are identified by using inducible forms of the TF. Imposing a gene expression constraint on the identification of direct target genes is important, because fewer than 10% of genes that are bound by a TF are regulated by that TF (Farnham, 2009). Genome-wide analysis identified 98 genes that are both bound by ABI3 and regulated following the induction of ABI3 activity, including genes encoding 2S seed albumins, 12S seed storage globulins, oleosins, and desiccation-related LEA proteins (Monke et al., 2012). Most of these target genes are generally expressed during the maturation phase, and they require abscisic acid for their activation, consistent with the observation that mutations in *ABI3* confers insensitivity to ABA (Koornneef et al., 1984). Analysis of the ABI3 target genes identified two DNA sequence motifs that are both overrepresented in the first 250 bp upstream of the transcription start site: a RY element that is known to be bound by ABI3 and a G-box motif. The G-box is part of a well-characterized ABA-responsive element (e.g., *ABRE*) motif that interacts with bZIP TFs. These finding are consistent with previous studies showing that ABI3 interacts with a bZIP TF to regulate the transcription of genes involved in maturation processes (Nakamura et al., 2001; Lara et al., 2003).

Target genes for another B3-domain maturation TF, FUS3, were identified from embryonic culture tissue overexpressing the *AGL15* gene that expresses *FUS3* constitutively (Wang and Perry, 2013). FUS3 target genes were enriched for maturation processes, and showed a 17% overlap with ABI3 target genes. The 5′ flanking regions of the FUS3 target genes were enriched for RY and G-box motifs. These studies confirmed on a genome-wide scale that there is at least partial redundancy in the functions of FUS3 and ABI3. FUS3 also directly regulates another B3-domain TF, VAL1, which along with VAL2 and VAL3, acts as repressors of the maturation network during seedling development (Suzuki and McCarty, 2008). FUS3 was also shown to regulate miRNA genes, including *miR156*, *miR160*, *miR166*, *miR169*, *miR369*, and *miR390*. Thus, FUS3 may be involved in controlling the shift from the morphogenesis to maturation phase given the proposed role of *miRNA156* in this transition.

Genetic and molecular studies place LEC1 at or near the top of the regulatory hierarchy controlling the maturation phase (Kagaya et al., 2005a; To et al., 2006). Analysis of genes that are bound and regulated by LEC1 identified two genes, *LEC1-LIKE* and *FATTY ACID BIOSYNTHESIS2*, which suggested a potential role for LEC1 in lipid biosynthesis and other maturation processes (Junker et al., 2010). Other direct target genes regulated by LEC1 are involved in auxin and brassinosteroid biosynthesis and signaling, light responses and transcription regulation. The studies also demonstrated an interaction between LEC1 and ABA signaling. For example, although LEC1 can bind to the 5′ flanking sequences of the *YUC10* gene that encodes an auxin biosynthetic enzyme in the absence of ABA, LEC1-induced *YUC10* expression is ABA dependent. Together, these results suggest that LEC1 plays an integrative role during plant development.

### GENOMICS OF ENDOSPERM DEVELOPMENT

Endosperm development is initiated with the fertilization of the central cell of the female gametophyte by a sperm cell and proceeds through three distinct stages in most angiosperms: syncytial, cellularization, and cellular (Olsen, 2004; Li and Berger, 2012). During the syncytial stage, the endosperm undergoes nuclear divisions without corresponding cell divisions, generating a syncytium of nuclei that each associates with a cytoplasmic region to form nuclear-cytoplasmic domains (Brown et al., 1999). This period of syncytial development is followed by cellularization in which cell walls form around nuclear cytoplasmic domains, beginning after the eighth nuclear divisions in *Arabidopsis*. Cellularization proceeds in a wave-like manner from the micropylar to the chalazal ends of the endosperm (**Figure 1A**). During the cellular stage, additional endosperm cells are formed through cytokinesis primarily at the periphery of the endosperm. Complex patterning of the endosperm is perhaps best exemplified by the *Brassicaceae*, including *Arabidopsis* and canola, in which three distinct endosperm subregions form corresponding to their positions within the seed: micropylar, peripheral, and chalazal (**Figure 1A**). These spatial domains are specified at the earliest stage of endosperm development in that their nuclear, cytoskeletal, and cytoplasmic characteristics and positions within the endosperm are distinguished by the fourth mitotic division (Brown et al., 2003). Depending upon the species, the endosperm remains largely intact throughout seed development as occurs in cereal grains, or it degrades as in *Arabidopsis*, canola, and soybean seeds.

### ENDOSPERM DOMAINS HAVE DISTINCT AND OVERLAPPING FUNCTIONS

Transcriptome analyses of the *Arabidopsis* endosperm have provided novel insights into the relationship between the micropylar, peripheral, and CZE subregions. Previous work using LMD to profile endosperm mRNA populations provided the first characterization of gene expression genome-wide in the micropylar, peripheral, and chalazal subregions (Belmonte et al., 2013). These studies showed that a small subset is expressed specifically in each endosperm subregion at virtually all stages of development, suggesting strongly that each subregion fulfills a unique function within the seed. In particular, the CZE has the largest number of genes that are expressed specifically in a single subregion of the seed and the most seed-specific genes among all subregions. Analyses of these CZE-specific genes showed that they encoded rate-limiting enzymes involved in the biosynthesis of the hormones gibberellic acid, abscisic acid, and cytokinin (Day et al., 2008; Belmonte et al., 2013), confirming the work of others who localized these enzymes to the CZE (Miyawaki et al., 2004; Lefebvre et al., 2006; Hu et al., 2008). Chalazal endosperm-derived abscisic acid, cytokinin, and gibberellic acid, respectively, are involved in controlling seed dormancy, endosperm cellularization, and growth of maternal tissues. Thus, the CZE may serve as a hub that supplies hormones to regulate developmental processes in developing seeds.

Analyses of the transcriptome datasets uncovered dominant patterns of gene activity for mRNAs that are involved in processes critical for seed development and that occur in all three endosperm domains and in the embryo. Clustering analyses identified a number of different gene sets that are expressed at early stages of seed development in the embryo and micropylar and PEN, but their expression in the CZE is delayed until the late developmental stages (Belmonte et al., 2013). One set encodes proteins involved in cytokinesis, consistent with the observation that embryo cells undergo cytokinesis concurrently with mitosis, whereas endosperm cellularization proceeds from the micropylar to the chalazal ends of the endosperm. Another set is involved in photosynthesis and carbon metabolism, a surprising result given that these processes were known to occur in the embryo but much less was known about their role in the endosperm. Additional analyses provided strong evidence that maturation processes occur not only in the embryo but also in all endosperm subregions. Together, these results emphasize a strong degree of overlap in gene expression programs between the embryo and endosperm regions of the seed.

### GENOMIC IMPRINTING AND THE CONTROL OF SEED SIZE

The endosperm has a profound influence on seed size. It has been shown or hypothesized that the size of the endosperm early in seed development, the timing of cellularization of endosperm cells, the provisioning of maternally derived nutrients from the endosperm to the embryo, and the influence of the endosperm on the proliferation and elongation of SC cells are major determinants in specifying seed size (Scott et al., 1998; Garcia et al., 2003, 2005; Melkus et al., 2009; Ohto et al., 2009). The endosperm influences seed size through parent-of-origin effects. Parent-of-origin effects are exemplified by genetic crosses between plants of different ploidy levels. Progeny from interploidy crosses that have an excess of maternal genomes (e.g., tetraploid female crossed with diploid male) produce seeds that are smaller than self-fertilized diploid plants, whereas plants with an excess of paternal genomes (e.g., diploid female by tetraploid male) produce larger seeds (Scott et al., 1998). The parental conflict theory has been proposed to explain the antagonistic influences of the mother and father. It is hypothesized that in polygamous organisms, the father will attempt to enhance the allocation of maternally derived resources specifically to his offspring to maximize their growth, whereas the mother will try to distribute resources equally to all offspring to equalize their growth (Haig and Westoby, 1989).

Parental influences on seed size are thought to be mediated by genomic imprinting. Imprinted genes are expressed following fertilization predominately from either the maternal or paternal alleles unlike the vast majority of genes that are expressed nearly equally from both alleles. Imprinted genes are thought to control resource allocation to the embryo and therefore support its growth. Consistent with this hypothesis, an imprinted gene has been shown to be involved in controlling maternal nutrient uptake and seed biomass (Costa et al., 2012). Imprinted genes have been identified using RNA sequencing experiments in which *Arabidopsis* plants of different ecotypes were crossed, and mRNAs from maternal and paternal alleles in the progeny were distinguished based on single nucleotide polymorphisms (Gehring et al., 2011; Hsieh et al., 2011; Wolff et al., 2011). These studies identified between 60 and 208 imprinted genes and showed that maternally expressed imprinted genes (MEGs) are more prevalent than paternally expressed imprinted genes (PEGs). Although these studies rarely identified any genes as being imprinted in the embryo, a recent study by Raissig et al. (2013) identified 11 MEGs and one PEG in the *Arabidopsis* embryo.

Genomic imprinting is regulated through epigenetic mechanisms involving DNA methylation and the Polycomb Repressive Complex 2 (PRC2). 5′-Methylcytosine in DNA is an epigenetic mark that is often associated with transcriptionally silenced genes, and PRC2 mediates gene silencing through the trimethylation of lysine 27 of histone H3 (H3K27me3, Kohler et al., 2012). To dissect the mechanisms regulating imprinted genes, Hsieh et al. (2011) and Wolff et al. (2011) analyzed the effects of mutations that cause defects in DNA methylation, DNA demethylation, and the PRC2 complex on gene imprinting. Collectively, their results showed that the DNA methylation status of MEGs correlated strongly with their imprinting. During female gametophyte development, the genome of the central cell, that is the maternal precursor of the endosperm, becomes hypomethylated globally due to the activity of DME, a DNA glycosylase that removes methylcytosine residues from DNA (Gehring et al., 2009; Hsieh et al., 2009). Hypomethylation of MEGs in the central cell results in the expression of maternal alleles of MEGs in the endosperm, whereas the paternal alleles retain their DNA methylation marks and remain silenced. The paternal alleles of some MEGs have also been shown to be silenced through the PRC2 pathway. By contrast, the paternal alleles of PEGs are active, but the maternal alleles are silenced predominately through the PRC2 pathway. These studies support the idea that demethylation of the maternal allelle of some PEGs is required to permit the gene to be silenced by the PRC2 (Weinhofer et al., 2010). Thus, a complex set of epigenetic regulatory mechanisms underlies genomic imprinting.

A potential causal link between parent-of-origin effects and endosperm size came from studies of 24 nucleotide p4 siRNAs in developing endosperm (Lu et al., 2012). p4 siRNAs, which in endosperm are derived specifically from the maternal genome, function in RNA-dependent DNA methylation to target specific loci for methylation (Mosher et al., 2009; Law and Jacobsen, 2010). p4 siRNAs primarily target transposable elements for DNA methylation. However, a significant fraction of genes are closely associated with transposons, and methylation of some of these transposons influences the expression of the linked gene. Genome-wide profiling of sRNAs in interploidy crosses of *Arabidopsis* showed that 24 nt siRNAs corresponding to specific genomic loci were strongly overrepresented in endosperm of seeds with a maternal genome excess relative to seeds with a paternal genome excess. Several of these loci corresponded to genes encoding AGL TFs, one of which has been shown to inhibit endosperm cellularization (Kang et al., 2008). These findings were interpreted to indicate that p4 siRNAs targeting AGL TFs are overrepresented in endosperm with a maternal genome excess, causing premature repression of the expression of *AGL* genes and precocious cellularization, resulting in a smaller seed. Together, these findings indicate a critical role for the endosperm in several aspects of seed development.

## GENOMICS OF SEED COAT DEVELOPMENT

Compared to the embryo and endosperm, the SC has received little attention at the genomics level. The maternally derived SC is responsible, in part, for the evolutionary success of the seed, and it plays an integral role in filling (Verdier et al., 2013), protection, and dispersal of seeds (Haughn and Chaudhury, 2005). The SC region, like the embryo and endosperm, can further be divided into subregions based on morphological and anatomical features. For example, in *Arabidopsis* and canola, the distal SC comprised the inner and outer integuments, undergoes dramatic anatomical transformations including cell expansion, changes in cell wall deposition, and anthocyanin and mucilage accumulation followed by programed cell death, all in preparation for seed dormancy. Conversely, the CZSC, located proximal to the funiculus, is found at the junction with the maternal plant. In seeds of legumes, like soybean, a total of six subregions have been identified: (i) endothelium, (ii) hour glass, (iii) palisades, (iv) parenchyma, (v) epidermis, and (vi) hilum. The hilum in soybean is considered to be similar in function to the CZSC in *Arabidopsis* and presents the first point of entry of material destined for filial seed compartments.

While the development and anatomy of the SC in oilseeds, such as *Arabidopsis*, soybean, and canola, have been extensively studied (Beeckman et al., 2000; Western et al., 2000; Windsor et al., 2000; Macquet et al., 2007; Young et al., 2008; Dean et al., 2011) there is remarkably little information about the genes and gene regulatory networks underlying this multicellular structure. Even less information is available about the genomics of SC development in emerging model crop systems. Of the few studies that have examined the SC at the genomics level (Jiang and Deyholos, 2010; Dean et al., 2011; Belmonte et al., 2013; Khan et al., 2014), data suggest the SC is more similar to maternal tissues than to the embryo or the endosperm. Despite the vast amount of data currently being generated and the different technologies being employed to study the SC, it is still unclear how many genes are active in each subregion and how those numbers change between species.

### TRANSCRIPTIONAL REGULATION IN THE SEED COAT

When comparing the SC to other seed regions, *Arabidopsis* is the best plant model studied to date. Hierarchical clustering of GeneChip data showed differences between each subregion of the SC. It is clear that global similarities and differences exist in the SC region compared to the embryo and endosperm and have likely evolved over time to protect the embryo and to adapt to environmental conditions (Debeaujon et al., 2000). Quantitative differences in gene activity within subregions of the SC provided insight into the biological processes underlying its development. Dominant patterns of gene expression were identified from comprehensive RNA profiling of *Arabidopsis* seed subregions. This analysis identified sets of genes that show spatial (between different subregions) and temporal (across seed development) differences in expression (Belmonte et al., 2013; Khan et al., 2014). Co-expressed gene sets were shown to represent biological processes associated with the development of SC color (Zhang et al., 2013), anthocyanin deposition (Debeaujon et al., 2003), and mucilage accumulation (Western et al., 2001), which have been extensively studied using forward genetic analyses. These studies revealed essential processes associated with the SC that are controlled by individual genes or small sets of genes, yet it was still unclear how all of these processes may be coordinated over the lifecycle of the seed.

Cellular processes that occur in the SC have been independently shown to be controlled by TFs belonging to MYB (Nesi et al., 2001; Penfield et al., 2001), HD-Zip (Johnson et al., 2002; Ishida et al., 2007), and MADS-Box (Nesi et al., 2002; Huang et al., 2011) families. Our comprehensive SC transcriptome analysis identified all of these TF mRNAs in a single analysis (Khan et al., 2014). Not only were all of these known regulators identified in our experiment, we also identified a number of possible gene targets responsible for cell fate specification, the accumulation of mucilage, the deposition of anthocyanin, flavonoid biosynthesis, and SC color.

### TRANSCRIPTIONAL REGULATION OF SEED COAT COLOR

Seed coat color is an agroeconomically important trait and is determined by the presence or absence of flavonoids, more specifically, proanthocyanidins. Flavanoids are secondary metabolites produced in plants derived from the phenylpropanoid pathway and are thought to have a number of functional roles, including photoprotection (Agati et al., 2013) and cellular signaling (Pourcel et al., 2013). Proanthocyanidins accumulate exclusively in the SC. When cells in the SC die, the proanthocyanidins oxidize and polymerize to form brown pigments that darken the seed. Mutants that have defects in proanthocyanidin production form a lighter colored or transparent SC (yellow/green). The yellow/green SC coloration is often associated with other desired agroeconomic traits such as thinner SCs, decreased fiber, and higher protein and oil contents (Simbaya et al., 1995; Lipsa et al., 2011; Jiang et al., 2013b). Proanthocyanidin deficient mutants do not appear to have any major physiological disturbances other than SC color; however, some evidence suggests the mutants may have diminished responses to abiotic/biotic stress (Pourcel et al., 2013), longevity, and germination (Dean et al., 2011; Jiang et al., 2013a).

Seminal work in the genetics and biochemistry of SC color in *Arabidopsis* revealed complex networks of genes and gene products responsible for this trait (Yu, 2013). In canola and soybean, genes that contribute to SC color are more difficult to identify genetically due to redundancies within the genome. RNA sequencing of brown- and yellow-coated *B. juncea* revealed three dihydroflavonol reductase genes and three anthocyanin reductase genes that were highly expressed in the brown-seeded variety with almost no detectable expression in the yellow-seeded variety (Liu et al., 2013a). The expression of three phenylpropanoid biosynthetic genes, ten flavonoid biosynthetic genes and four regulatory genes were studied using qRT-PCR at seven developmental stages in yellow- and brown-seeded *B. napus*. Two propanoid biosynthetic genes (*PHENYLALANINE AMMONIA LYASE*, *TRANS-CINNAMATE 4-MONOOXYGENASE*), two flavonoid biosynthetic genes (*TRANSPARENT TESTA4, 6*), five anthocyandin/proanthocyandin biosynthetic genes (*3,4-DICHLOROPHENOL GLYCOSYLTRANSFERASE 2, TRANSPARENT TESTA3, 10, 12, 18*), and three TFs (*TRANSPARENT TESTA8, TRANSPARENT TESTA GLABRA1, 2*) had different expression patterns in yellow seeds (Qu et al., 2013). Further, eleven quantitative trait loci mediating SC color and fiber content were identified using high-density SNP arrays in canola (Liu et al., 2013a). Together genomics studies of SC color provide new targets for improving desirable traits, such as seed oil quality, and highlight the genetic complexity of SC color (Liu et al., 2013b). The analysis and identification of new QTLs combined with RNA sequence data should provide the information needed to design improved breeding strategies.

### TRANSCRIPTIONAL REGULATION IN THE CHALAZAL SEED COAT

While the distal SC has been the primary focus of numerous functional studies, the CZSC has not been studied in the same detail. Bioinformatic analysis of CZSC mRNA populations uncovered a number of transport processes that showed dynamic programs of activity across development. These processes had not been described previously because of the inaccessibility of the CZSC within the seed for experimental analysis. For example, genes associated with phloem unloading including *SUCROSE-PROTON SYMPORTER 2* (*SUC2*) and a complement of *SWEET* genes encoding sucrose eﬄux transporters, amino acid transport genes including *BIDIRECTIONAL AMINO ACID TRANSPORTER 1* (*BAT1*), *AMINO ACID PERMEASE 2* (*AAP2*), water transport genes encoding tonoplast intrinsic proteins (TIP1;1), and plasma membrane intrinsic protein are all expressed in the CZSC. These findings support the hypothesis that transport processes are enriched in the CZSC. Co-expression networks generated from transcriptome data provided insight into the regulation of these transport processes. A putative G-box regulated network controlling water and sugar transport in the developing seed through bZIP TFs, including bZIP25, bZIP28, and LRL1 (Khan et al., 2014). Functional characterization of these transcriptional regulators predicted to be associated with CZSC function presents a new avenue of targeted seed improvement through modification of maternally derived subregions.

## IDENTIFYING REGULATORY NETWORKS REQUIRED TO PROGRAM THE *Arabidopsis* SEED

To better understand the underlying transcriptional mechanisms required to program the seed, an integrative systems biology approach should be applied that incorporates molecular and computational biology. First, large-scale datasets are required for such an approach, and excellent sources of seed genomic data are available at databases such as GEO and NCBI as discussed previously. However, mining this data effectively requires the development of more advanced and user-friendly tools that are available to a broader scientific audience through online databases. Tools from the BioArrayResource ^8^, Genevestigator ^9^, and The *Arabidopsis* Information Resource ^10^ are all excellent resources for genomics-based data including but not limited to whole seed, seed region, and seed subregion datasets. In addition, the seedgenenetwork.net database houses whole seed, seed region, and seed subregion transcriptome, sRNA, and DNA methylome datasets from *Arabidopsis* and soybean. Although usability of online tools continues to improve, it remains difficult to identify genes with key roles in seed development with these online tools.

Using high-resolution seed datasets from *Arabidopsis* (Le et al., 2010; Belmonte et al., 2013; Khan et al., 2014), we developed a user-friendly bioinformatics program to identify transcriptional circuits from large-scale datasets at every stage of the seed lifecycle ^11^. We identified genes, focusing our attention on TFs that are predicted to control biological processes across developmental time or that are specific to a seed subregion, including the embryo proper, micropylar endosperm, CZE, or the distal and CZSCs. The transcriptional module analysis is based on the association of a specific set of co-expressed genes with their enriched Gene Ontology terms, known DNA sequence motifs, metabolic processes, and TF families and presents the user with possible gene targets regulating biological processes within the seed.

**FIGURE 4:**
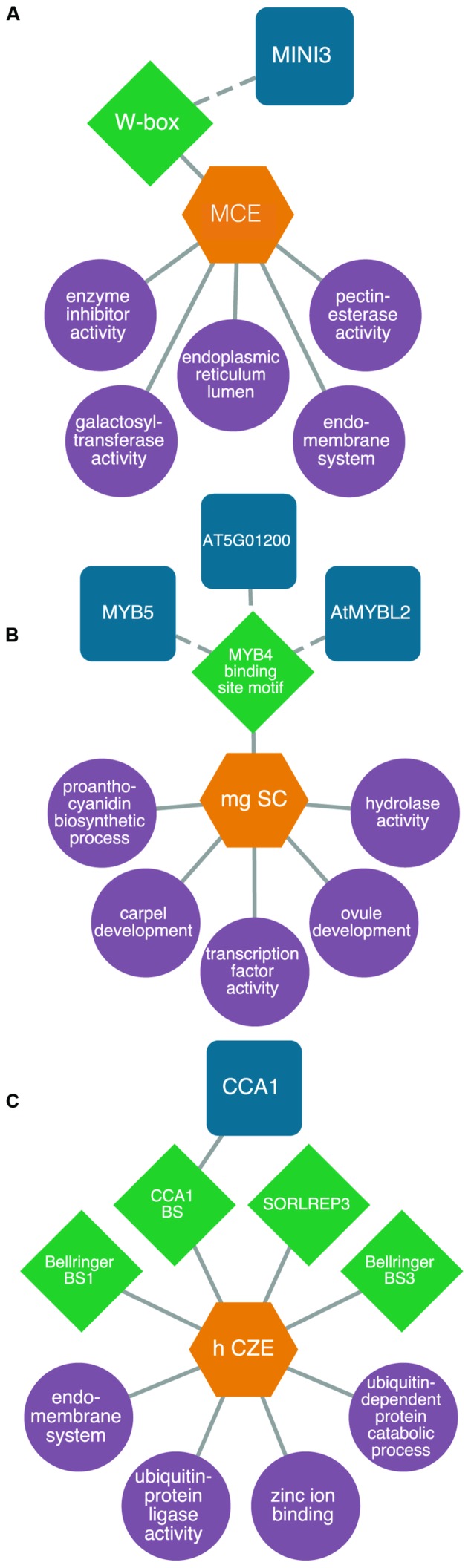
**Predictive transcriptional circuits in subregions of the *Arabidopsis* seed**. **(A)** *MINISEED3* (*MINI3*)-W-box transcriptional circuit in the micropylar endosperm (MCE) regulating processes like the endomembrane system. **(B)** A *CIRCADIAN CLOCK ASSOCIATED1* (*CCA1*) module in the chalazal endosperm (CZE) of heart-stage seeds. **(C)** A *MYB* transcriptional module in the mature green (mg) seed coat (SC) predicted to control processes like proanthocyanidin metabolism and ovule and carpel development. TFs (blue squircles) are predicted (dashed lines) or known (solid lines) to bind to DNA sequence motifs (green diamonds) within the 1 kb upstream region of the transcription start site in genes associated with enriched (*P* < 0.001, hypergeometric distribution) GO terms (purple circles) within patterns of co-expressed gene sets (orange hexagons). All networks are modified from Belmonte et al. (2013).

For example, we identified a transcriptional module consisting of genes expressed specifically in the micropylar endosperm and that are enriched for the WRKY DNA sequence motif in their 5′ flanking regions. Our model predicts *MINISEED3* to control processes associated with the endomembrane system in the early stages of seed development. While *MINISEED3* has previously been shown to localize to the micropylar endosperm (Luo et al., 2005), the model allows us to predict gene targets of this TF which were previously unknown (**Figure 4A**). We also studied a putative transcriptional network underlying the CZE. Up until recently, genetic information about this under-studied subregion was lacking. However, through our integrative bioinformatics approach we identified a putative *CIRCADIAN CLOCK ASSOCIATED1*-regulated transcriptional circuit controlling ubiquitin-dependent protein catabolic processes (**Figure 4B**). Within the SC, we identified a number of regulators that have been previously associated with SC development, allowing a high degree of confidence in our predictive transcriptional modules (**Figure 4C**). The TRANSPARENT TESTA GLABRA complex is implicated in the regulation of flavonoid biosynthesis, and several MYB TFs (including MYB5) are implicated in the regulation of mucilage biosynthesis and the differentiation of the outer integuments (Khan et al., 2014).

While this type of data analyses has been used successfully to identify existing transcriptional circuits, the real power of this approach lies in the identification of unknown interactions and prediction of the biological processes controlled by a TF. One of the caveats to this method is that a well-annotated genome must be available as a reference. Thus, one of the challenges in emerging crop systems will be the annotation of genomes for which genomics research is still in its early stages. While we are beginning to understand some of the molecular mechanisms underlying the development and properties of different seed subregions and regions, the interconnectedness of these transcriptional circuits will remain a priority in the effort to elucidate the complex regulatory pathways responsible for seed development. The spectacular increase in genomic resources applicable to the seed will enable a more comparative approach to uncover and study both conserved and unique transcriptional circuits among related seed species such as the *Brassicaceae* or the *Leguminosae.* Current efforts are directed at implementing and developing computational programs to identify gene regulatory networks for important crop species like canola and soybean. The ability to predict transcriptional circuits in cell and tissue types previously thought to be inaccessible within the seed provides unprecedented insight into the regulation of biological processes over developmental time.

## IDENTIFICATION OF TFs ESSENTIAL FOR SEED DEVELOPMENT

Analysis of putative gene regulatory networks is an excellent way to identify possible regulators of seed development. However, experimental validation and functional characterization of the TFs are required to validate the network. Identification of essential seed genes is a cumbersome task yet remains a priority for those interested in studying seed biology and genomics. While research has focused on essential seed genes that when mutated cause a seed lethal phenotype, other mutant phenotypes may result in defects in metabolic pathways or biochemical processes, cellular development, morphology, or other more subtle molecular phenotypes. Through our work, we have identified a number of region- and subregion-specific TFs; however, the vast majority of mutant alleles of these regulators failed to show a seed lethal phenotype (Le et al., 2010; Belmonte et al., 2013). Thus, the function of most subregion-specific TF mRNAs discovered in our work remains unknown.

Much has been learned about the seed through the use of forward genetics. Forward genetics involves generation of random mutations within an organism through radiation-, chemical-, or insertion-induced mutagenesis followed by screening for an aberrant phenotype. Systems for phenotyping mutants are becoming increasingly automated (Fiorani and Schurr, 2013), and NGS strategies are being used to map the mutation site in what is being referred to as “fast-forward” genetics (Schneeberger and Weigel, 2011). Through forward genetics, an extensive collection of *Arabidopsis* T-DNA mutants is available through the SALK Institute (Alonso et al., 2003), and a database of essential seed genes has been established at seedgenes.org (Meinke et al., 2008) and the *Arabidopsis* Biological Resource Center.

As we continue to characterize the seed genome, forward genetics becomes increasingly ineffective as the likelihood of discovering previously uncharacterized mutants decreases. Molecular tools such as RNA interference and over-expression lines have provided researchers with important information about their genes of interest. However, new genome editing techniques utilizing the CLUSTERED REGULARLY INTERSPACED SHORT PALINDROMIC REPEATS (CRISPR)/CRISPR-Associated System (CAS; Xie and Yang, 2013), Transcription Activator-Like Effector Nucleases (TALENs; Christian et al., 2013), and Zinc Finger Nucleases (ZFNs; Zhang et al., 2010; de Pater et al., 2013), are becoming popular alternatives to classical mutagenesis. Unlike the previous approaches that relied solely on chance, emerging technologies provide an efficient means to achieve targeted mutagenesis and target multiple alleles simultaneously (Curtin et al., 2011). In addition, there is the potential for targeting non-coding regions of the genome to elucidate regulatory functions of nucleic acid sequences (Gaj et al., 2013). Of these systems, the most recent to emerge is the CRISPR/CAS system. Unlike ZFNs and TALENs that rely on complicated protein–DNA interactions, the CRISPR/CAS system uses guiding RNAs and simple base pairing between the RNA construct and target site. In addition, this technology has the ability to perform multiple genome edits by targeting more than one location simultaneously (Cong et al., 2013). This technology is also proving to have several additional practical applications, such as the modification of gene expression *in vivo* through gene fusion to transcriptional activation or repression domains (Bikard et al., 2013) or for the labeling of individual chromosomal loci (Chen et al., 2013). Taken together, the ability to manipulate transcriptional networks and fine-tune gene expression would prove valuable tools for the molecular dissection and engineering of seeds.

## FUTURE DIRECTIONS

It is an exciting time to study the underlying mechanisms of seed development through genomics. The complex morphological and metabolic transformations of the seed lend themselves to intensive genomic interrogation. While seminal work dissecting cells, tissues, and organs of both *Arabidopsis* and soybean seeds has revealed an incredible abundance of information, there are still pressing questions when it comes to the coordination and regulation of seed development at the cellular and tissue levels. To answer these questions seed biologists are using modern sequencing strategies. The incredible amount of information produced by these technologies is overwhelming, and the information extracted from these analyses will only continue to improve as we perfect the chemistries and foster new collaborations with mathematics, statistics and computer science. These in-depth analyses yield significant information about the transcriptional circuitry underlying complex tissue systems responsible for the development of the seed. Moreover, identification of transcriptional regulators from large-scale datasets will provide the necessary starting point for research focusing on improving seeds.

To achieve these goals, plant biologists are coupling cutting-edge technologies that are capable of dissecting or isolating individual cells and tissues of the seed with sequencing platforms. In addition to mRNA profiling, LMD has been coupled to genomics strategies such as bisulfite sequencing to study global changes in DNA methylation marks, degradome sequencing to study miRNA cleavage sites, and ChIP sequencing to identify protein/TF DNA interactions during seed development. DNA sequencing, bisulfite sequencing, RNA and small-RNA sequencing, degradome sequencing, ChIP sequencing, and CLIP sequencing (protein–RNA interactions) each provide a piece to the developmental puzzle, and sophisticated integrative computational analyses will be required to put all of the pieces together. Thus, the development of integrative computational tools to analyze complex and possibly disparate datasets in all plants will remain a major challenge for the scientific community.

Despite the tremendous advances in genomics-focused research including NGS platforms and the continuing reduction in the cost and production of high-resolution datasets, functional characterization of genes responsible for seed development, especially in emerging model systems, remains a challenge. Functional testing and characterization of the biological information derived from the billions of data points that sample the dynamic biological processes underlying seed development will take decades using current molecular biology tools. Thus, high-throughput functional characterization of genes and gene products remains a top priority for plant biologists. There are four areas of seed genomics and its application that we suggest need to be targeted to further improve our understanding of the seed: (i) update and curate small- and large-scale genomics data in publicly available databases; (ii) implement user-friendly data analysis pipelines and educate scientists on how to use them effectively; (iii) profile and characterize the genomes of emerging models important for global crop production and development; (iv) functionally characterize every gene responsible for plant traits relevant to sustainable agriculture.

## CONCLUSION

Current advancements in seed genomics are illuminating the genetic forces driving seed development. It is now possible to identify most of the genes responsible for guiding seed development in every cell, tissue, and organ throughout the seed lifecycle. Together, modern breeding strategies that include information derived from genomics-based research will provide the necessary tools to improve seeds: seeds with improved nutritional value, that can endure adverse environmental conditions, or one that can withstand biological attack. Our dependence on seeds for food, fuel, and other resources means seed improvement research through genomics will continue to have a significant impact on global biosustainability.

## Conflict of Interest Statement

The authors declare that the research was conducted in the absence of any commercial or financial relationships that could be construed as a potential conflict of interest.

## Footnotes

1 http://www.ncbi.nlm.nih.gov

2 http://www.ncbi.nlm.nih.gov/geo

3 http://www.ncbi.nlm.nih.gov/sra

4 http://aralip.plantbiology.msu.edu/

5 http://www.mirbase.org

6 http://mirtarbase.mbc.nctu.edu.tw

7 http://www.cassava-genome.cn/mirfans

8 http://www.bar.utoronto.ca

9 http://www.genevestigator.com

10 http://www.arabidopsis.org

11 http://seedgenenetwork.net/presentation#software
